# Comparative analysis of two kinds of garlic seedings: qualities and transcriptional landscape

**DOI:** 10.1186/s12864-023-09183-x

**Published:** 2023-02-24

**Authors:** Penghui Ai, Jundong Xue, Yifei Zhu, Wenchao Tan, Yifei Wu, Ying Wang, Zhongai Li, Zhongya Shi, Dongru Kang, Haoyi Zhang, Liwen Jiang, Zicheng Wang

**Affiliations:** State Key Laboratory of Crop Stress Adaptation and Improvement, Plant Germplasm Resources and Genetic Laboratory, Kaifeng Key Laboratory of Chrysanthemum Biology, School of Life Sciences, Henan University, Jinming Road, Kaifeng, 475004 Henan China

**Keywords:** *Allium*, Garlic seedlings, Blanched garlic seedlings, Transcriptome, Darkness, Light

## Abstract

**Background:**

Facility cultivation is widely applied to meet the increasing demand for high yield and quality, with light intensity and light quality being major limiting factors. However, how changes in the light environment affect development and quality are unclear in garlic. When garlic seedlings are grown, they can also be exposed to blanching culture conditions of darkness or low-light intensity to ameliorate their appearance and modify their bioactive compounds and flavor.

**Results:**

In this study, we determined the quality and transcriptomes of 14-day-old garlic and blanched garlic seedlings (green seedlings and blanched seedlings) to explore the mechanisms by which seedlings integrate light signals. The findings revealed that blanched garlic seedlings were taller and heavier in fresh weight compared to green garlic seedlings. In addition, the contents of allicin, cellulose, and soluble sugars were higher in the green seedlings. We also identified 3,872 differentially expressed genes between green and blanched garlic seedlings. The Kyoto Encyclopedia of Genes and Genomes analysis suggested enrichment for plant-pathogen interactions, phytohormone signaling, mitogen-activated protein kinase signaling, and other metabolic processes. In functional annotations, pathways related to the growth and formation of the main compounds included phytohormone signaling, cell wall metabolism, allicin biosynthesis, secondary metabolism and MAPK signaling. Accordingly, we identified multiple types of transcription factor genes involved in plant-pathogen interactions, plant phytohormone signaling, and biosynthesis of secondary metabolites among the differentially expressed genes between green and blanched garlic seedlings.

**Conclusions:**

Blanching culture is one facility cultivation mode that promotes chlorophyll degradation, thus changing the outward appearance of crops, and improves their flavor. The large number of DEGs identified confirmed the difference of the regulatory machinery under two culture system. This study increases our understanding of the regulatory network integrating light and darkness signals in garlic seedlings and provides a useful resource for the genetic manipulation and cultivation of blanched garlic seedlings.

**Supplementary Information:**

The online version contains supplementary material available at 10.1186/s12864-023-09183-x.

## Background

The progression of industrialization and urbanization, the increase in the human population, and the sharp decrease in land and water resources have led to the necessity for the efficient use of limited agricultural resources as well as improved crop yield and quality. Facility agriculture and facility cultivation are modern agricultural production methods that can solve the problem of low efficiency of resource transformation caused by the deterioration of traditional agricultural production modes and the agricultural ecological environment [1]. Facility cultivation is mostly implemented for research on annual production dynamics and the breeding of new varieties for ornamentals, vegetables, fruits, medicinals, grain, and economic crops [1–6]. Indeed, facility cultivation provides a unified and tightly controlled environment in which to explore the regulatory mechanisms governing plant growth and development, and to assess fertilizer requirements, photosynthetic capacity, stress tolerance, and other research, with the ultimate goal to reach higher yields and produce high-quality plants [2, 3]. The ability to precisely control the growth environment in a protected cultivation setting is also more conducive to an in-depth study of plant physiology and molecular biology. Research on light quality and light intensity has always been a hot topic in plants, and facility cultivation has played a key role [7–15].

The *Allium* genus includes garlic (*A. sativum*), onion (*A. cepa*), chive (*A. tuberosum*), leek (*A. porrum*), and shallot (*A. cepa*), all economically important vegetables, herbs, and ornamentals. Facility cultivation is widely used in *Allium* [10, 16–19]. Therefore, a better understanding of the effects of light quality and light intensity on the growth and quality of *Allium* will help improve yield and quality. Garlic is an important vegetable crop from the Amaryllidaceae family [19, 20]. Blanched garlic seedlings and green garlic seedlings are widely cultivated and consumed as a vegetable in China due to their high nutritional value, strong bactericidal activity, and cancer prevention, detoxification, intestinal cleansing, blood sugar reduction, and cardiovascular and cerebrovascular disease prevention activities [19, 21]. Blanched garlic seedlings are produced by exposing garlic seedlings to a blanching culture regimen consisting of transfer to a low-light or darkness environment. Blanching culture is one facility cultivation mode that promotes chlorophyll degradation, thus changing the outward appearance of crops, and improves their flavor; notably, several vegetable crops such as garlic, celery (*Apium graveolens*), and hotbed chives are cultivated in this manner [22, 23]. Several studies have focused on the flavor, nutritional contents, and active compounds accumulating in blanched garlic seedlings [19, 21]. However, the molecular consequences of blanching culture on the development of garlic seedlings are understudied. Understanding the effects of blanching cultivation on the growth and development of garlic seedlings and the biosynthesis of nutrients and active compounds may guide the application of garlic yellowing cultivation.

For vegetables with health-promoting properties, the levels of bioactive compounds are an important contributor to quality. Allicin is the key bioactive molecule in garlic, garlic seedlings, and blanched garlic seedlings and is derived from alliin via alliinase [19, 20, 24–26]. The alliin biosynthetic pathway has been extensively characterized in garlic [27–31]. Indeed, many functional genes, including those encoding gamma-glutamyl transpeptidase (*AsGGT*), flavin-containing monooxygenase (*AsFMO*), gamma-glutamylcysteine synthetase (*AsGSH*), and phytochelatin synthase (*AsPCS*), have been identified along the allicin biosynthetic pathway in garlic [19, 29–31]. Additionally, genome sequencing and transcriptome deep sequencing revealed 60 alliinase genes in various garlic tissues [19]. The synthesis of allicin is affected by many conditions, such as high and low mineral nutrition [32–38], varying light intensity, light quality, and photoperiod conditions [39, 40], different garlic varieties [41–43], and in response to various temperatures [39]. Therefore, it is crucial to study these mechanisms affecting allicin synthesis.

Yield is also an important factor in the growth of garlic seedlings. Previous studies showed that when grown under darkness or low-light conditions, seedlings rapidly elongate their hypocotyl (for dicots) or mesocotyl (in monocots) to rapidly reach the light above the soil surface [44] by modulating the expression of many genes [45–50].

Since garlic’s ability to generate real seeds was lost thousands of years ago, only vegetative propagation is used today [51, 52]. Viruses spread from vegetative propagules to the other, and other media further transmit the viruses from infected to healthy plants. As a result, plants suffer 25 to 50% yield losses and quality degradation [53]. The long history of co-evolution between garlic and its pathogenic microorganisms has gradually formed a complex network that balances plant defenses against pathogens and plant growth [54–56]. It would be interesting to study how light and darkness signals participate in this complex network.

In recent years, RNA-seq has been applied to global transcriptome analyses of garlic seedlings and flower development [19, 26, 57–59], secondary metabolite biosynthesis [19, 26], and stress tolerance [26, 58] in various tissues. This approach uncovered the molecular mechanisms underlying various processes and empowered the discovery of many candidate genes and the expression characteristics of important genes across various conditions [19]. For instance, 36 alliinase genes were shown to be constitutively expressed in various tissues, while another 21 alliinase genes were dynamically expressed in developing bulbs, the *WUSCHEL*-related homeobox gene Asa7G00799.1 was expressed exclusively in bulbs, while *AsGSH1b*, *AsGSH2*, *AsPCS1*, *AsFMO1*, and *AsGGT2* were constitutively expressed [19]. Garlic has a relatively large genome compared to those of other eukaryotes [19]. The high- quality reference genome illustrates the complex gene regulatory networks captured by RNA-seq. Fortunately, whole-genome fine-mapping of desirable garlic traits has been established using next-generation sequencing [19], providing a reliable reference genome for transcriptome analysis.

While many studies have focused on the flavor, nutritional contents, and active compounds of garlic seedlings, they have to date largely been limited to green seedlings. The molecular effects of blanching culture on the development of garlic seedlings are understudied. Here, we combined physiological characteristics with RNA-seq analysis to identify differentially expressed genes (DEGs) involved in metabolic pathways in two culture systems. The identification of DEGs in garlic seedlings and blanched garlic seedlings may provide new genetic resources for this species. Understanding the effects of yellowing cultivation on the growth and development of garlic seedlings and the biosynthesis of nutrients and active compounds may be directly used to guide the cultivation conditions of blanched garlic seedlings.

## Results

### Comparison of green and blanched garlic seedlings

**Fig. 1:**
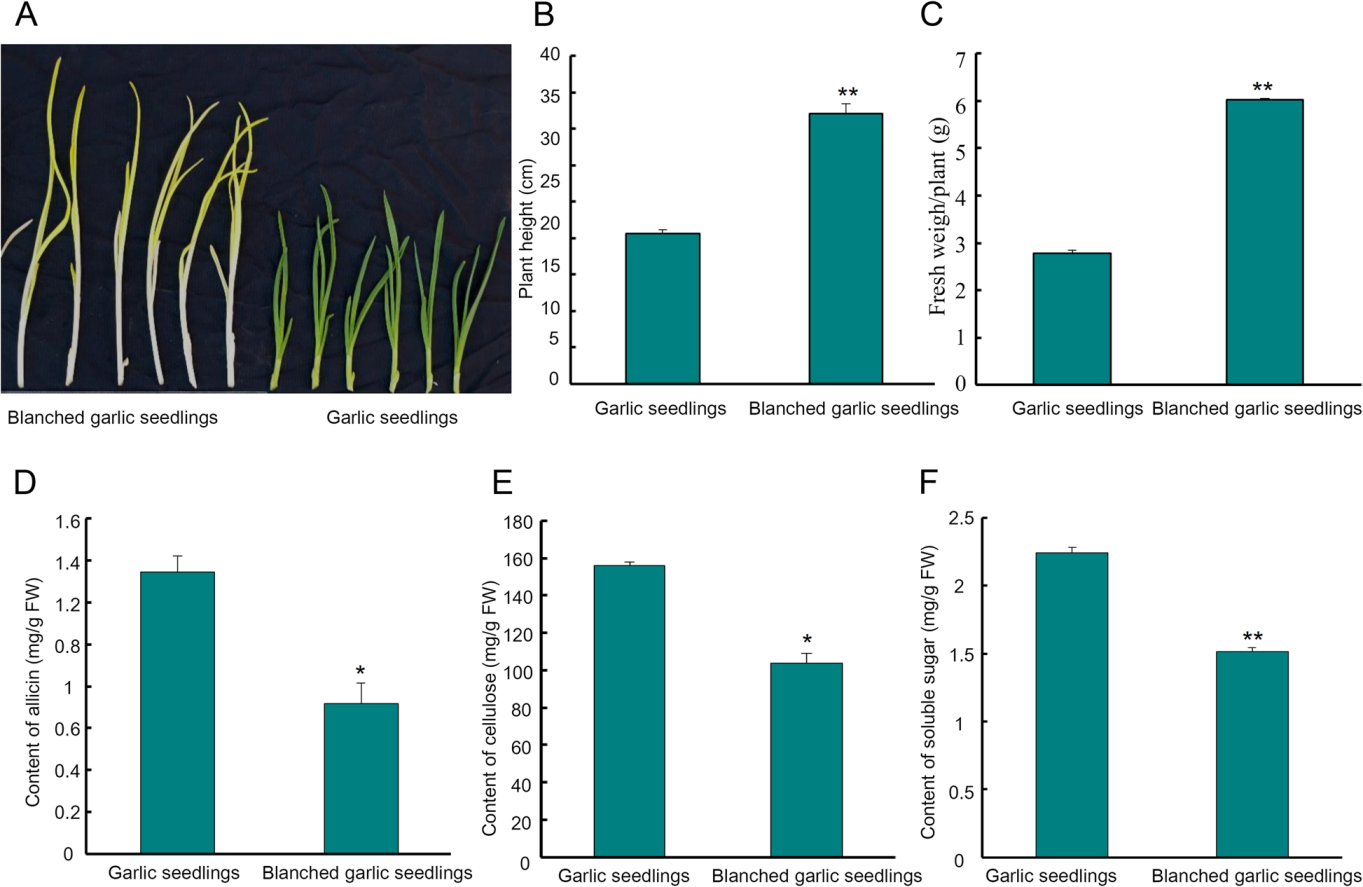
Comparative characterization of garlic seedlings and blanched garlic seedlings. **A：** Phenotype of garlic seedlings and blanched garlic seedlings; **B**: Comparative plant height of garlic seedlings and blanched garlic seedlings; **C**: Comparative fresh weight of garlic seedlings and blanched garlic seedlings; **D**: Difference of total allicin content in garlic seedlings and blanched garlic seedlings; **E**: Difference of total cellulose content in garlic seedlings and blanched garlic seedlings; **F**: Difference of total soluble sugar content in garlic seedlings and blanched garlic seedlings. The vertical bars indicate the mean ± SE of three biological replicate. Statistical significance was determined by Student's t tests; significant differences (*P < 0.05, **P < 0.01) are indicated by different numbers of *

To investigate the effects of blanching culture on the growth of garlic seedlings, followed by a 14-day simulated blanching culture treatment. Blanched garlic seedlings were taller and accumulated more fresh weight compared to seedlings maintained under light/dark conditions (Fig. 1A, B, C). In addition, the contents of allicin, cellulose, and soluble sugars clearly decreased in the blanched seedlings relative to their green siblings (Fig. 1D, E, F), demonstrating the substantial influence of the light environment on the growth of garlic.

### RNA-seq analysis of green and blanched garlic seedlings

To better understand the molecular mechanism behind the dark or shade response of garlic seedlings, we performed an RNA-seq analysis on three biological replicates per growth condition. We obtained from 41.8 to 51.7 million raw reads per biological replicate with an average read length of 100 bp (Table 1). After trimming adapters and removing low-quality reads and virus read contaminants, we retained 134.6 million clean reads across the six sequencing libraries, with the percentage of reads with a quality score of Q20 (sequencing error rates lower than 1%) or higher being above 98.3%. Of these clean high-quality reads, between73.95% and 81.9% mapped to the *Allium sativum* reference genome across the six samples [19], with 68.35%–75.30% of reads mapping to a single genomic location and another 5.6%–6.6% mapping to multiple locations. We normalized transcript levels as FPKM (fragments per kilobase of exon per million mapped fragments) [60] before assessing the correlations among biological replicates. The resulting Pearson’s correlation coefficients for FPKM values per genes among the biological replicates for each growth condition were higher than 0.93 (Fig. S1). Moreover, the biological replicates collected from the same type of samples clustered together (Fig. S2), as evidenced by the correlation matrix and principal component analysis (PCA) [61]. Overall, we concluded that the RNA-seq results are reproducible and reliable.

**Table 1:** Summary of the sequence data from RNA sequencing

Sample	Raw Reads	Clean Reads	Q20 (%)	Mapped Reads	Mapping Ratio (%)	Multiple Mapping Ratio (%)
blanched garlic seedling-1	41,843,572	20,921,786	98.41	30273259	72.35	5.96
blanched garlic seedling-2	51,752,60	25,876,300	98.31	35370867	68.35	5.56
blanched garlic seedling-3	42,240,026	21,120,013	98.38	31808710	75.3	6.26
garlic seedling-1	42,733,924	21,366,962	98.37	30264933	70.82	6.15
garlic seedling-2	47,441,532	23,720,766	98.35	32634712	68.79	5.74
garlic seedling-3	43,100,372	21,550,186	98.51	32373885	75.11	6.23

### Identification and analysis of DEGs

**Fig. 2:**
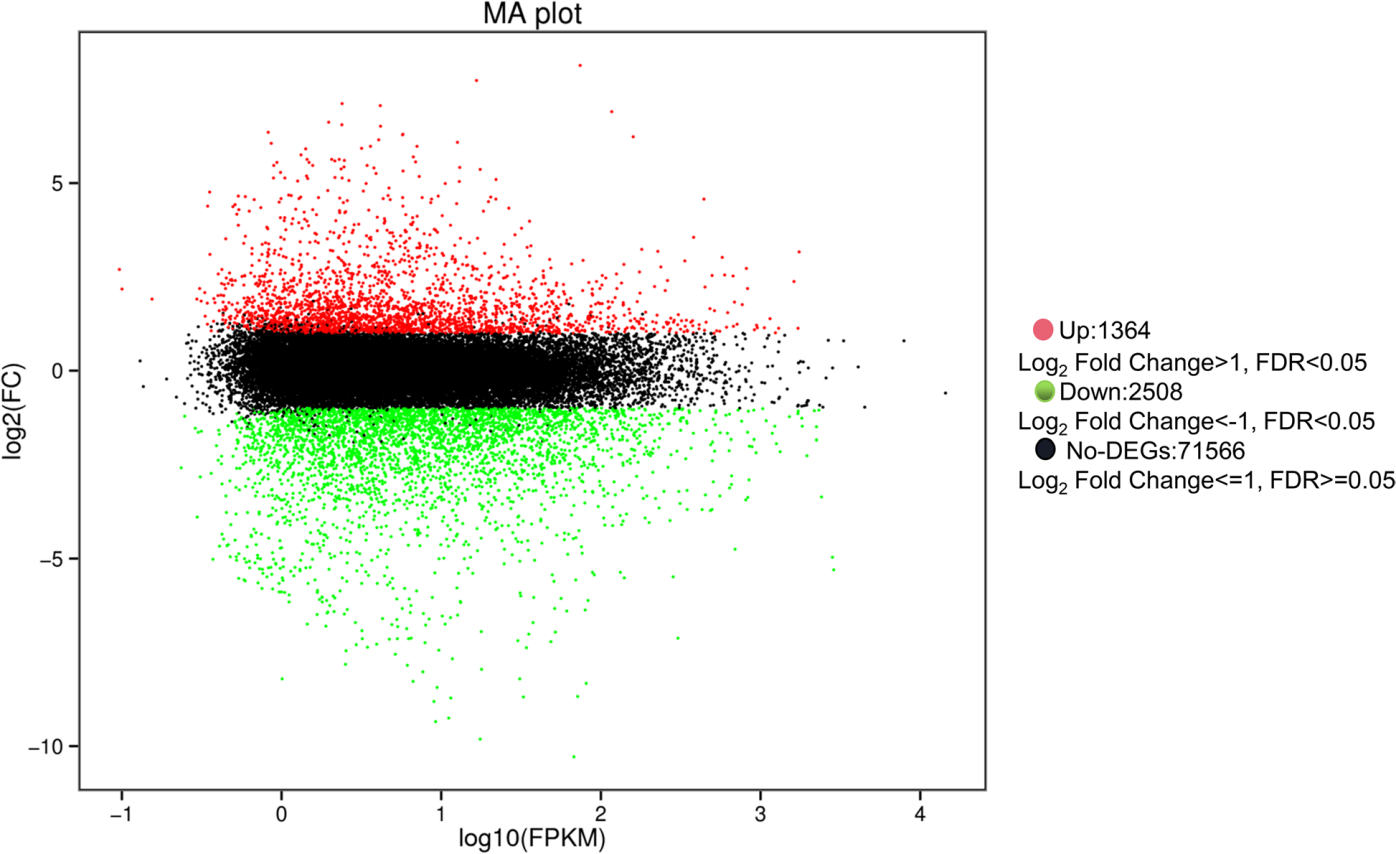
The expression profile of DEGs in garlic seedlings and blanched garlic seedlings

We identified differentially expressed genes (DEGs) between green and blanched garlic seedlings with the selection criteria *q*-value ≤ 0.05 and |Log_2_(FC)|≥ 1 using DEGseq2 software. We thus obtained 3,872 DEGs between the two groups, with 1,364 upregulated genes and 2,508 downregulated in blanched seedlings relative to green seedlings (Fig. 2). All DEGs are listed in Table S1.

**Fig. 3:**
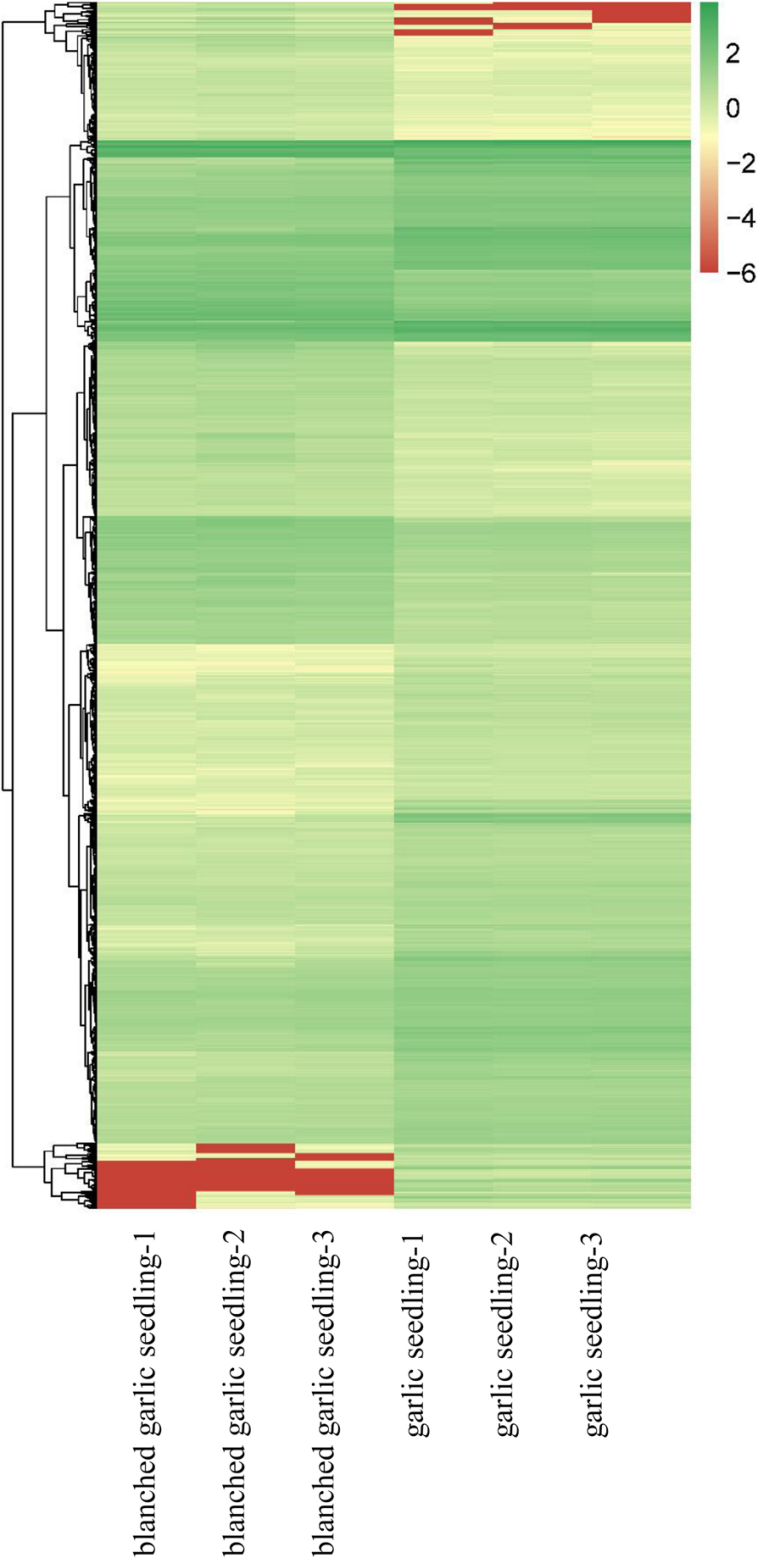
Hierarchical cluster analysis of 3872 DEGs based on the log (FC) of gene expression. The color gradient from low (green) to high (red) represents relative levels of gene expression. The numbers in the scale bar stand for the Z-score of gene expression

We subjected all 3,872 DEGs to hierarchical clustering, which roughly grouped them into five classes (Fig. 3). We determined that 78 DEGs are specifically expressed in green seedlings, including many genes primarily involved in five eggNOG classes: amino acid transport and metabolism; carbohydrate transport and metabolism; lipid transport and metabolism; secondary metabolite biosynthesis, transport, and catabolism; and transcription (Table S1). Conversely, six DEGs were specifically expressed in blanched seedlings, but belonged to different pathways (Table S1).

### GO and KEGG enrichment analysis of DEGs

**Fig. 4:**
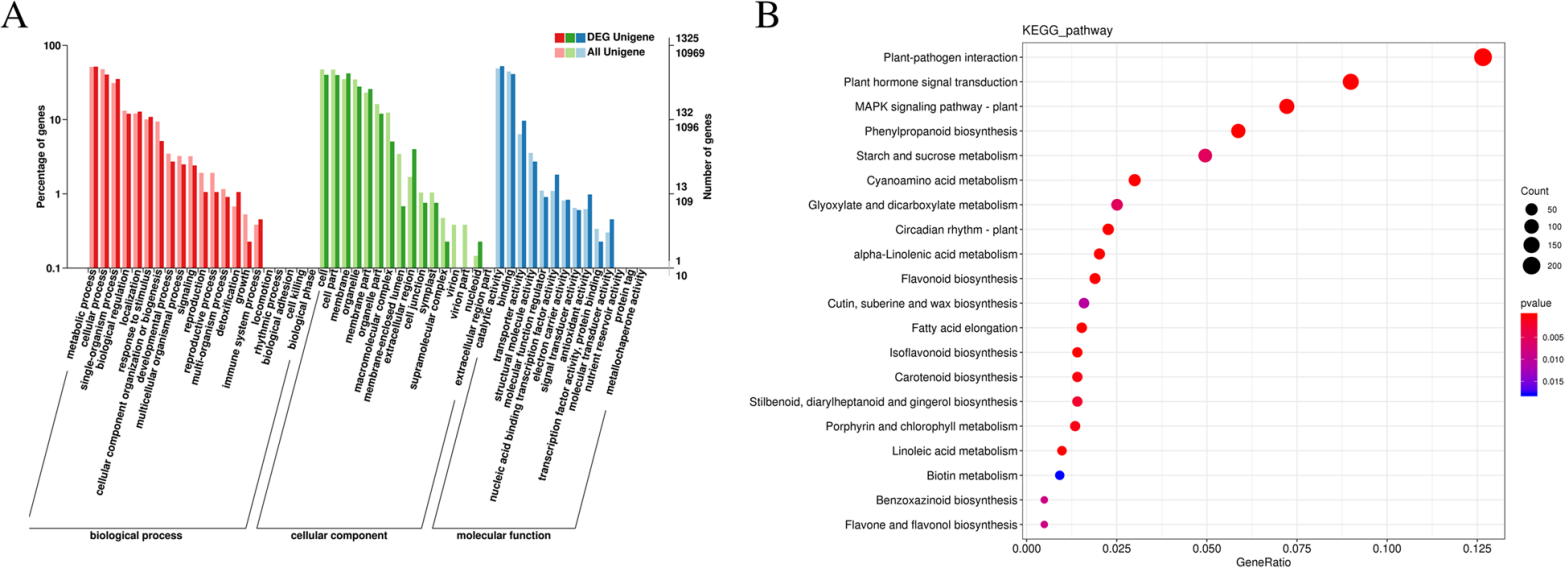
GO terms and KEGG pathways prominently enriched DEGs in garlic seedlings and blanched garlic seedlings [63–65]. **A：** GO terms prominently enriched DEGs in garlic seedlings and blanched garlic seedlings; **B**: KEGG pathways prominently enriched DEGs in garlic seedlings and blanched garlic seedlings. The abscissa is GeneRatio, that is, the proportion of genes annotated in the entry to the number of differentially expressed genes [63–65]

We then classified the DEGs on the basis of GO annotations using the R package *Cluster Profile* [62]. The 3,872 DEGs were enriched in 45 GO terms across the categories biological process, molecular function, and cellular component. Of these 45 GO terms, DEGs were enriched by a factor of over 100 for 21 GO terms over their genome-wide distributions (Table S2), with eight for cellular components, nine for biological processes, and four for molecular functions. “Metabolic process (GO:0,008,152)”, and “cellular process (GO:0,009,987)” were significantly enriched in the biological process. Major enriched molecular functions included catalytic activity (GO:0,003,824) and binding (GO:0,005,488). Among cellular components, cell (GO:0,005,623), membrane (GO:0,016,020), membrane part (GO:0,044,425), and cell part (GO:0,044,464) were enriched. Furthermore, the enriched GO terms from the garlic seedlings and blanched garlic seedlings were compared based on their biological processes (Fig. 4A).

**Fig. 5:**
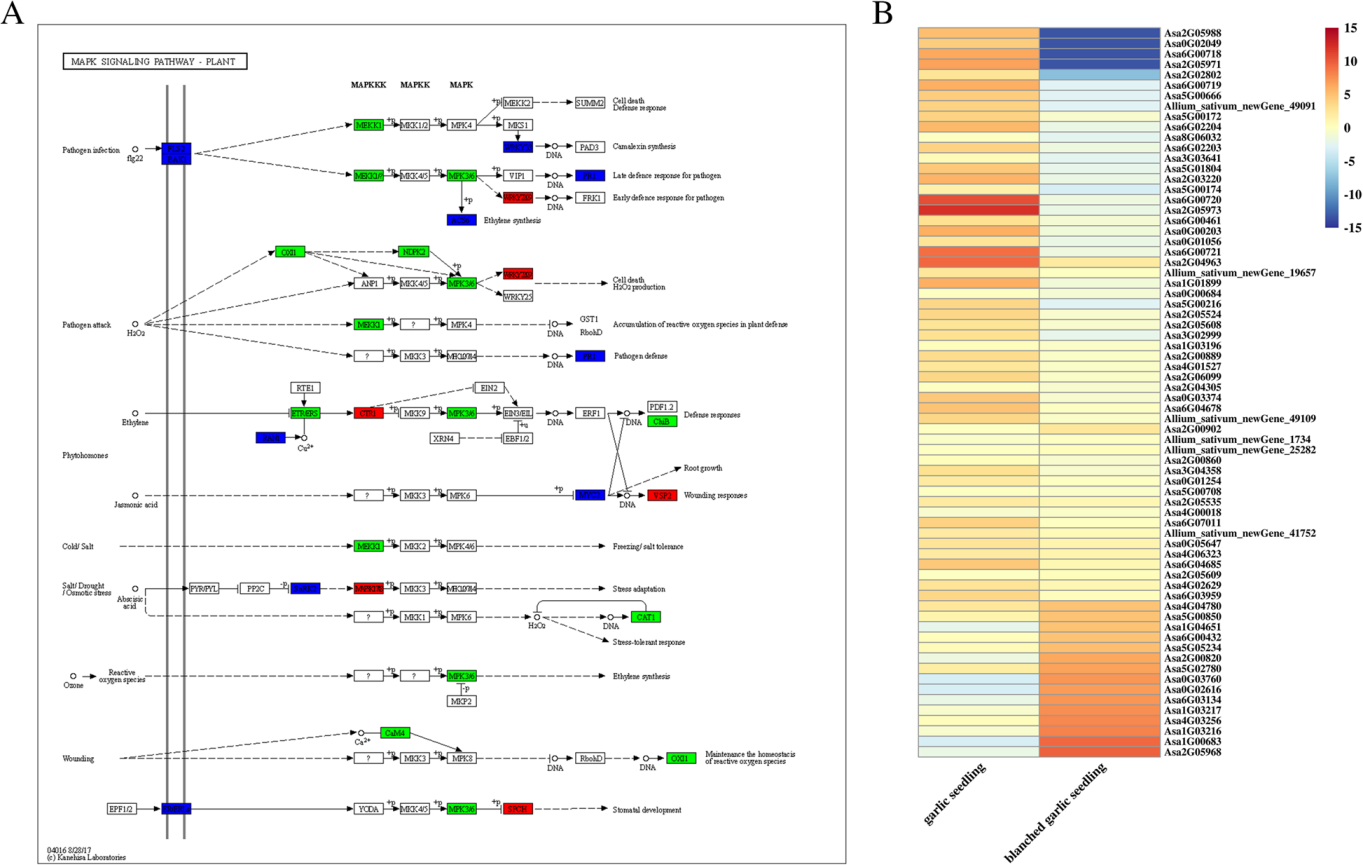
Differentially expressed transcripts involved in “MAPK signaling pathway-plant (ko04016)” pathway. **A**: Exhibition of “MAPK signaling pathway-plant (ko04016)” pathway, some DEGs are shown in the pathway; **B**: Heat map of gene expression patterns involved in “MAPK signaling pathway-plant (ko04016)” pathway obtained from KEGG [63–65]. Heat maps were drawn according to log2FPKM in garlic seedlings and blanched garlic seedlings. The rows and columns in the heat maps represent samples and genes id, respectively. Red and blue represent the highest and lowest level of expression

To identify the metabolic pathways in which DEGs participate, we employed KofamKOALA to search all DEGs against the KEGG database [63–65]. We anchored all 3,872 DEGs to KEGG Orthology (KO) terms and established an enrichment of 1,029 DEGs among 20 pathways (Fig. 4B, Table S3). Notably, we observed the greatest enrichment for the pathways plant–pathogen interaction (ko04626, 207 DEGs), plant hormone signal transduction (ko04075, 147 DEGs), and Mitogen-activated protein kinases (MAPK) signaling pathway-plant (ko04016, 118 DEGs). Furthermore, the three pathways linoleic acid metabolism (ko00591), isoflavonoid biosynthesis (ko00943), carotenoid biosynthesis (ko00906), photosynthesis—antenna proteins (ko00196) and photosynthesis (ko00195) only include DEGs downregulated in blanched seedlings relative to green garlic seedlings (Tables S1 and 3). Mitogen-activated protein kinases (MAPKs) have been implicated in development and stress responses, including defense and resistance against pathogens [66]. We determined that most garlic genes related to the ko04016 pathway are upregulated in green garlic seedlings compared to blanched seedlings (Table S4, Fig. 5).

### Identification of GO terms associated with the regulation of growth

**Fig. 6:**
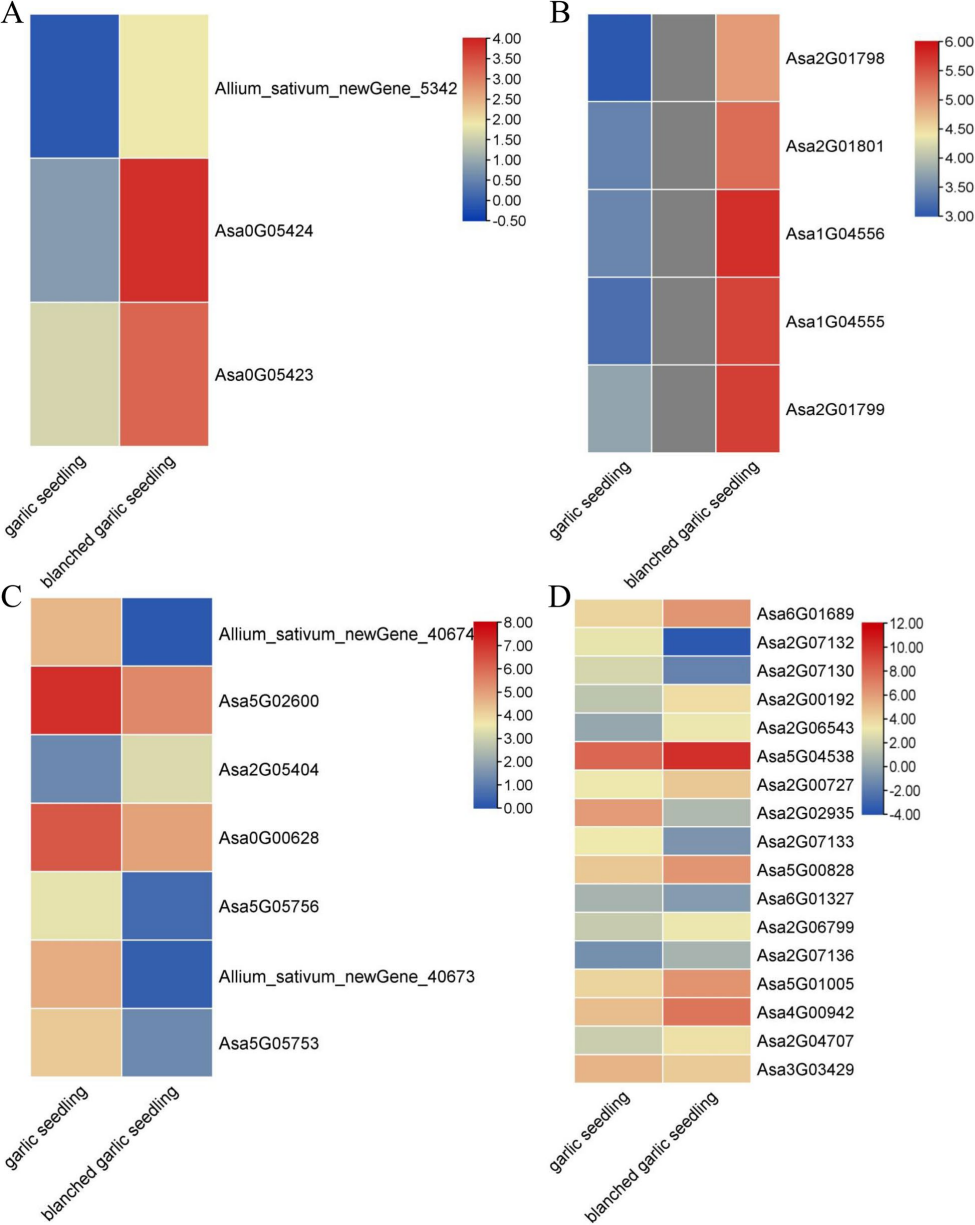
Heat maps of GO terms involved in the regulation of growth related genes in garlic seedlings and blanched garlic seedlings. Heat maps were drawn according to log2FPKM in garlic seedlings and blanched garlic seedlings. The rows and columns in the heat maps represent samples and genes id, respectively. Red and blue represent the highest and lowest level of expression. **A：** “positive regulation of organ growth” GO terms related genes; **B**: “regulation of monopolar cell growth” GO terms related genes; **C**: “regulation of leaf development” GO terms related genes; **D**: “cell wall biogenesis” GO terms related genes

The GO annotation results revealed several enriched GO terms related to growth among the DEGs, including regulation of leaf development; cell wall biogenesis; regulation of monopolar cell growth; and positive regulation of organ growth. Key genes, such as *LONGIFOLIA* and *ARGOS*, were upregulated in blanched seedlings compared to green seedlings (Table S5, Fig. 6A, B). In the GO term regulation of leaf development, seven genes were significantly downregulated in blanched seedlings (Table S5, Fig. 6C). Several DEGs were also enriched for the GO term cell wall biogenesis, including 11 upregulated and 6 downregulated genes in blanched seedlings (Table S5, Fig. 6D).

### Identification of DEGs involved in the biosynthesis of secondary metabolites

Green and blanched garlic seedlings contain many compounds: isoflavonoids, flavonoids, carotenoids, benzoxazinoids, flavones, flavonols, allicin, cutin, suberine, and wax, which contribute to their survival or are beneficial to human health [19, 25, 67–71]. Accordingly, we focused on DEGs involved in the KEGG pathways isoflavonoid biosynthesis; phenylpropanoid biosynthesis; flavonoid biosynthesis; carotenoid biosynthesis; benzoxazinoid biosynthesis; flavone and flavonol biosynthesis; and cutin, suberine, and wax biosynthesis (Table S3), which were significantly enriched in green seedlings. Isoflavonoids, flavonoids, flavones, flavonols, benzoxazinoids, cutin, suberine, and wax are common secondary metabolites that help plants adapt to environmental stress. All these metabolite biosynthesis pathways comprised 96 genes, of which 76 were significantly downregulated and 20 were significantly upregulated in blanched seedlings relative to green seedlings (Table S6). Phenylpropanoids are the first substrate for the biosynthesis of many secondary metabolites. In agreement with the importance of this biosynthetic pathway, many DEGs showed an enrichment with phenylpropanoid biosynthesis, with 25 upregulated genes and 71 downregulated in blanched seedlings relative to green seedlings (Table S6). Of these, 23 genes belonged to the carotenoid biosynthesis pathway and were significantly downregulated in blanched seedlings (Table S6).

**Fig. 7:**
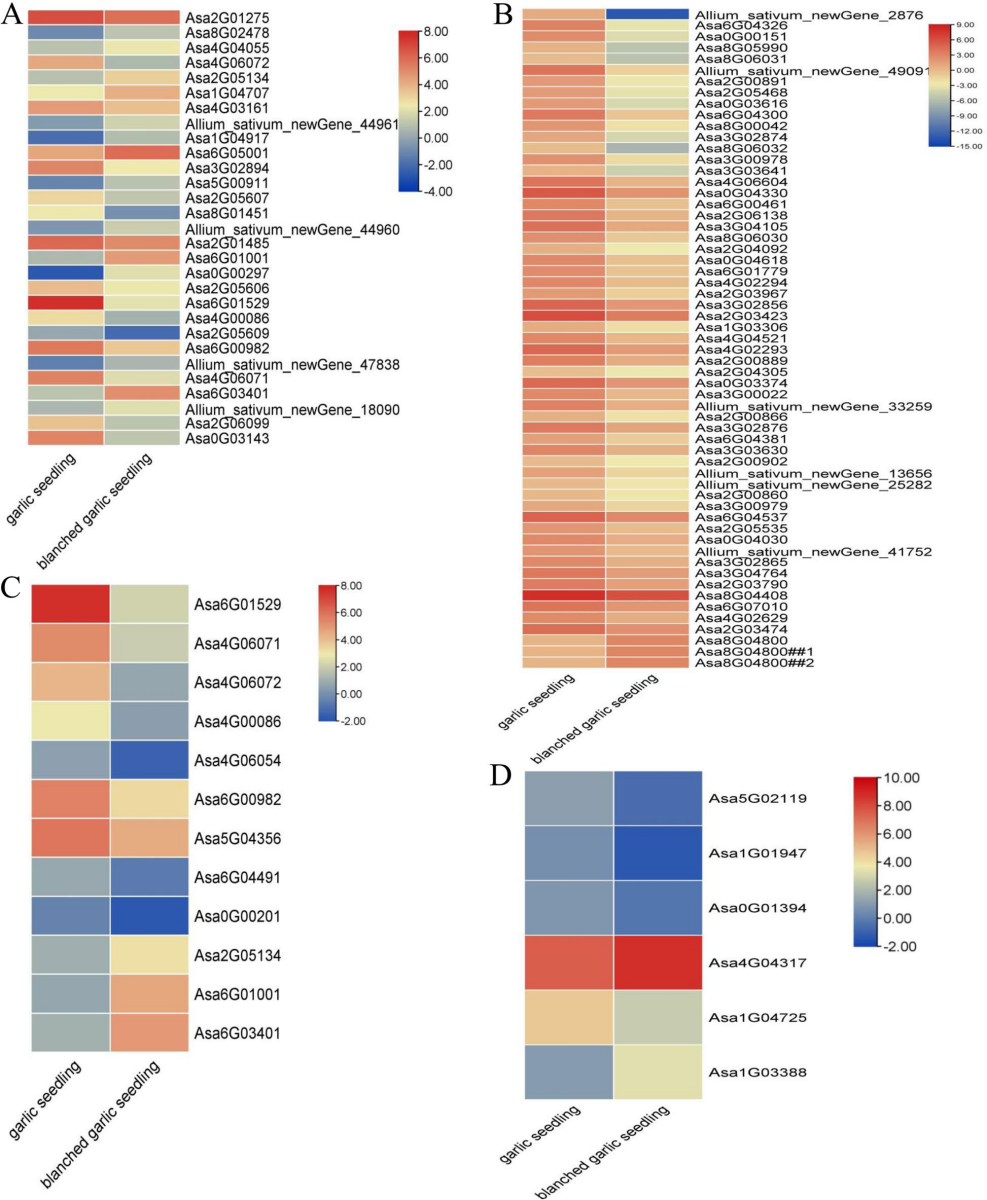
Heat maps of allicin biosynthesis related genes in garlic seedlings and blanched garlic seedlings. Heat maps were drawn according to log2FPKM in garlic seedlings and blanched garlic seedlings. The rows and columns in the heat maps represent samples and genes id, respectively. Red and blue represent the highest and lowest level of expression **A**: sulfur-related GO terms related genes; **B**: serine-related GO terms related genes; **C**: cystein-related GO terms related genes; **D**: alliin lyase genes related genes

Allicin biosynthesis involves its precursors (glutatione, glycine, serine, cysteine and sulfur) a series of hydrocarboxylation, alkylation, and oxidation reactions [19, 72–74]. We thus screened the DEGs for those that may be involved in allicin biosynthesis, resulting in the identification of 14 sulfur-related GO terms including 14 upregulated and 15 downregulated genes in blanched seedlings relative to green seedlings (Table S7, Fig. 7A). We also identified seven serine-related GO terms that include many DEGs, including 3 upregulated and 63 downregulated genes in blanched seedlings relative to green seedlings (Table S7, Fig. 7B). We also noticed four cysteine-related GO terms mapping to six DEGs, two upregulated and four downregulated genes in blanched seedlings relative to green seedlings (Table S7, Fig. 7D). Specifically looking for alliin biosynthetic genes, we identified 12 alliin lyase genes as being differentially expressed between the two groups of seedlings, with three genes upregulated and nine downregulated in blanched seedlings (Table S7, Fig. 7C).

**Fig. 8:**
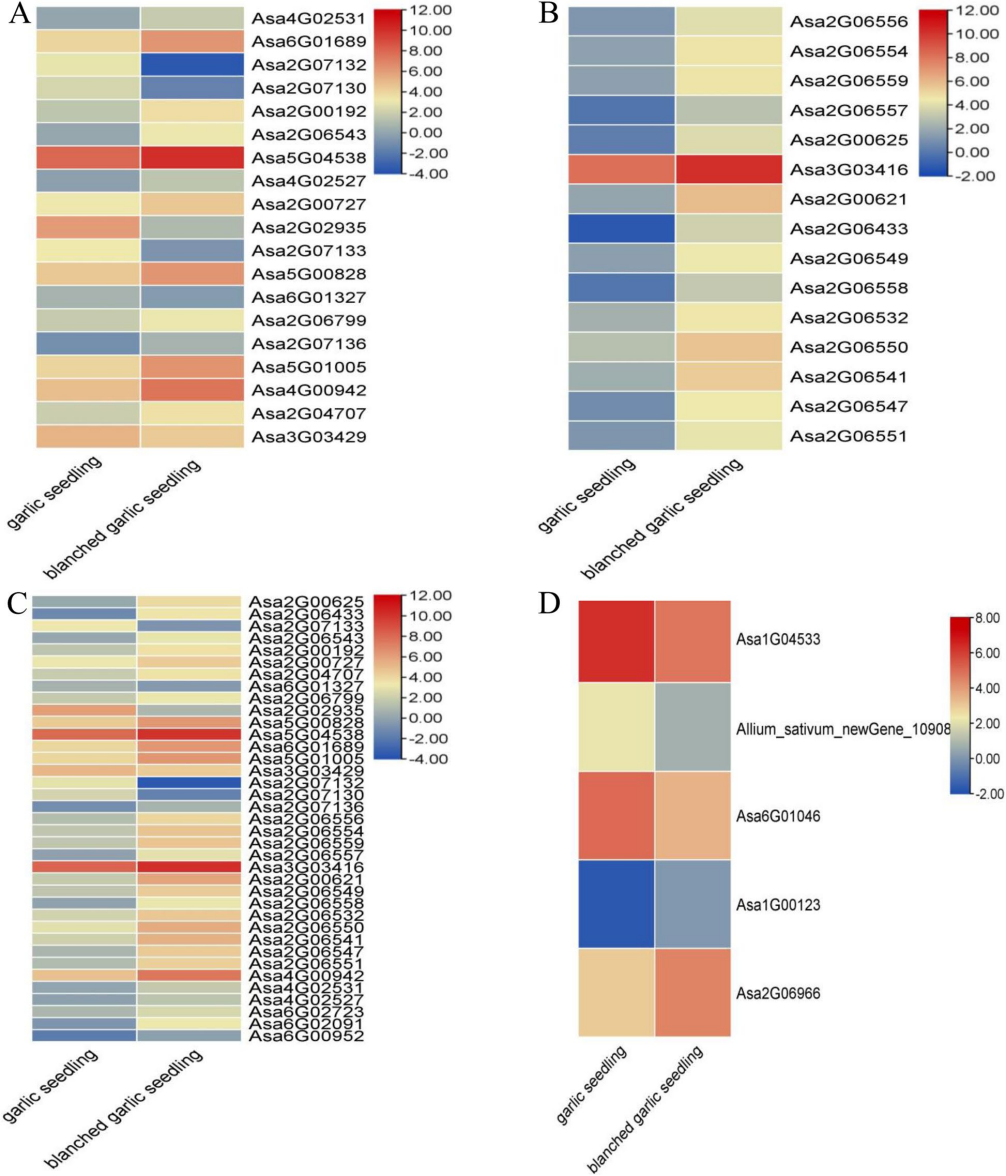
Heat maps of cellulose and hemicellulose biosynthesis and metabolism related genes in garlic seedlings and blanched garlic seedlings. Heat maps were drawn according to log2FPKM in garlic seedlings and blanched garlic seedlings. The rows and columns in the heat maps represent samples and genes id, respectively. Red and blue represent the highest and lowest level of expression. **A：** Xyloglucan metabolic process related genes; **B**: Cellular gulucan metabolic process related genes; **C**: Cellulose metabolism related genes; **D**: Cellulose biosynthesis related genes

Cellulose and hemicellulose contents of a vegetable or fruit directly affect how crisp they are [75]. Moreover, after enzymatic digestion, the metabolites contained within vegetables and fruits become more bioavailable for human nutrition, such as the xylose polymer xylooligosaccharide [76–79]. In this work, we screened for DEGs that might be involved in cellulose biosynthesis and metabolism, which established that three *endoglucanase* genes, 28 *xyloglucan endotransglucosylase/hydrolase* genes, and two *Altered Xyloglucan* genes were upregulated in blanched seedlings, while six *xyloglucan endotransglucosylase/hydrolase* genes were downregulated in blanched seedlings when compared to green seedlings (Table S8, Fig. 8), suggesting that cellulose metabolism is faster in blanched garlic seedlings than in garlic seedlings. This observation was consistent with the measured cellulose contents in the two sets of samples (Fig. 1E). Regarding cellulose biosynthesis, we noticed two upregulated cellulose synthase genes in blanched seedlings, while one cellulose synthase gene and two *COBRA* genes were downregulated in blanched seedlings relative to green seedlings (Table S8, Fig. 8). *COBRA* genes that encode additional proteins that are required for proper cellulose synthesis and orientation [80].

### Transcription factors play important roles in blanched garlic seedlings

**Fig. 9:**
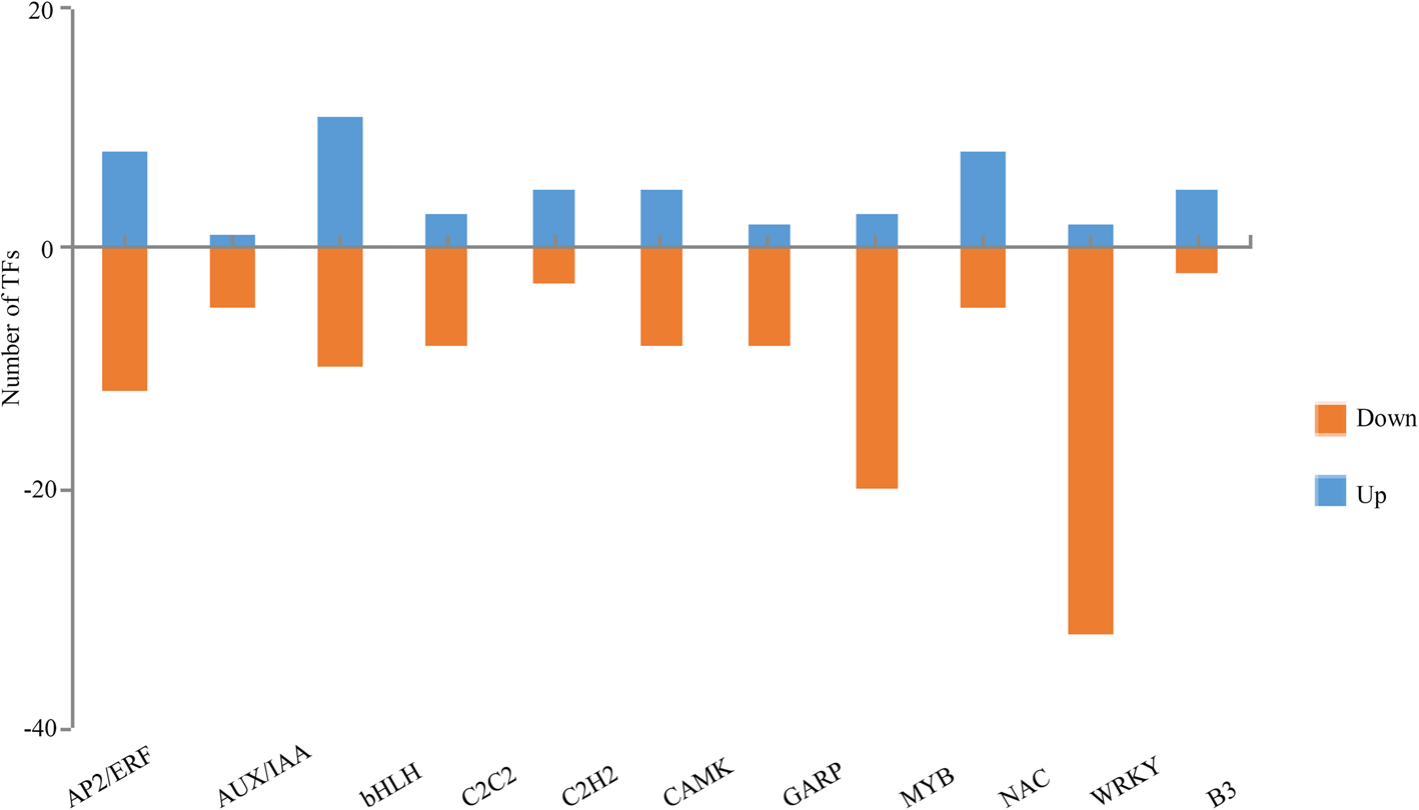
Determination of the top 11 transcription factor families in garlic seedlings and blanched garlic seedlings. Blue represents the up-regulated TFs, yellow represents the down-regulated TFs, and the vertical axis shows the number of TFs

We further explored the transcript levels of transcription factor (TF) genes to better understand the regulatory networks in blanched and green garlic seedlings. We observed that 80 and 145 TF genes belonging to 34 transcription factor families are upregulated and downregulated, respectively, in blanched seedlings compared to green seedlings in our RNA-seq dataset. The families with the highest number of encoding DEGs were APETALA2 (AP2; 20, 8.9%), basic helix-loop-helix (bHLH; 21, 9.3%), MYB (23, 10.2%), and WRKY (34, 15.1%) TFs. Figure 9 shows the number of upregulated and downregulated genes encoding the top 11 transcription factor families between blanched and green seedlings. A few TF genes were upregulated in blanched seedlings and appeared to be differentially expressed between the two sets of garlic seedlings; thus, they may play a crucial role in the observed differences in seedling development (Table S9, Fig. 9).

### Validation of RNA-seq analysis by RT-qPCR

To determine the reliability of DEGs obtained from RNA-seq analysis, we randomly selected 10 DEGs participating in different biological pathways for RT-qPCR validation, including *Alliin lyase*, *Allene oxide synthase*, and *HEC1* (Table S10), using *GAPDH* as the reference gene. We observed a very high and positive correlation (*R*^2^ = 0.963) between RT-qPCR and RNA-seq results (Fig. S3). This result unambiguously confirms the reliability of the DEGs obtained from RNA-seq analysis in this study.

## Discussion

Garlic is the most economically important member of the *Allium* genus and is rich in medicinal properties [19, 21]. The popularization of facility agriculture and facility cultivation for garlic farming has made light intensity and quality important factors affecting crop yield and quality [10–14, 17, 18, 81]. There has been much research into how light intensity and quality affect the growth and development physiological and phenotypic aspects of garlic and other *Allium* species [10–14, 17, 18, 21], but how their influences molecular networks remains unclear.

In this study, we showed that blanched garlic seedlings grow taller than garlic seedlings (Fig. 1A, B, C). However, blanched seedlings accumulated lower contents of allicin and cellulose compared to green seedlings (Fig. 1D, E). We employed RNA-seq to explore the effects of blanching culture on garlic seedling development. We then identified key genes and biological pathways enriched in KEGG and GO terms to comprehensively analyze the effects of blanching culture on gene expression and the underlying regulatory networks.

### Growth and developmental regulation of blanched and green garlic seedlings

In the dicot Arabidopsis (*Arabidopsis thaliana*), light, shade, and darkness act as signals to modulate the activity of the COP/DET/FUS (CONSTITUTIVE PHOTOMORPHOGENIC/DEETIOLATED/FUSCA) signaling complex and the transcription factors PHYTOCHROME INTERACTING FACTORs (PIFs) and affect apical hook development and hypocotyl elongation [82, 83]. In agreement with these observations, we showed here that blanched garlic seedlings are taller and accumulate more biomass than green garlic seedlings (Fig. 1). Interestingly, one *AsaPIF* gene was upregulated and one *AsaHY5* gene was downregulated in blanched seedlings (Table S1). Homeobox domain (HD) TFs in maize (*Zea mays*) may play both negative and positive roles in the regulation of axillary bud development [48]. Notably, we identified eight upregulated and four downregulated genes encoding HD TFs in blanched seedlings relative to green seedlings (Table S9). A similar opposite expression pattern was also reported for HD TF genes in response to shade in maize and Arabidopsis [50, 84].

Light, shade, and darkness all act as signals to regulate phytohormone (auxin, gibberellins [GAs], ethylene, and brassinosteroids [BRs]) levels and signaling pathways to affect plant development and morphology [47, 85]. In this study, genes associated with the GO term plant hormone signal transduction were differentially expressed between blanched and green garlic seedlings. Most DEGs involved in auxin signaling (encoding auxin-responsive proteins, auxin efflux carriers, and SAUR proteins) were upregulated in blanched seedlings. However, most DEGs involved in GA (encoding DELL protein), ethylene (encoding the ethylene-responsive transcription factor WRI1), and BR (encoding BR-insensitive 1-associated receptor kinase [BAK1]) signal transduction pathways were downregulated in blanched seedlings. Moreover, seven *YUCCA* (*YUC*) genes (auxin biosynthesis genes) were upregulated in blanched seedlings, while GA, ethylene, and BR biosynthesis genes were downregulated in blanched seedlings (Table S11). Our findings were also consistent with previous reports in other species [47, 85], suggesting that the growth of garlic seedlings under black or light conditions may be dependent on phytohormone signaling.

In this study, we also identified five *LONGIFOLIA1-like* genes and two *ARGOS-B* genes, which were all upregulated in blanched seedlings (Fig. 6). Previous studies have indicated that *LONGIFOLIA* genes and *ARGOS-B* regulate cell elongation in plants [86, 87]. Cell expansion and remodeling are the primary causes for the increase in plant organ size [88–90]. Additionally, most expansin, xyloglucan endotransglucosylase/hydrolase, and xylosidase genes were upregulated in blanched seedlings relative to green seedlings, in agreement with their greater height (Fig. 8). This result suggested that these genes may be involved in the development of garlic seedlings under dark conditions.

Furthermore, many genes involved in the KEGG pathway porphyrin and chlorophyll metabolism (ko00860), carotenoid biosynthesis (ko00906) and photosynthesis (ko00195) were upregulated in green seedlings, in accordance with their phenotypes (Fig. 1A, Table S1).

### Allicin biosynthesis

Most health issues worldwide are caused by cardiovascular complications (atherosclerosis, arterial thrombosis, and deep venous thrombosis) and neoplastic development [91, 92]. Some currently used synthetic anticoagulant drugs and free radical scavengers have various side effects [93, 94]. Therefore, functional foods such as fruits, vegetables, and teas have been well documented to play a substantial role in prevention and adjuvant therapy for some chronic diseases [95, 96]. Garlic has a long history as a spice and folk medicine whose root, bulb, leaf, and sprout extracts elicit diverse antibiotic, antineoplastic, antithrombotic, hypoglycemic, antioxidant, and anti-hypertensive biological functions [19, 25, 97]. These beneficial effects are mainly attributed to the production of secondary metabolites rich in sulfur [66, 98, 99]. Therefore, improving the accumulation and activity of secondary metabolites in garlic and garlic derivatives has become a hot topic of research [19, 20, 25, 26, 98].

Allicin is the key secondary metabolite and bioactive molecule in garlic [19, 20, 25–27]. Allicin biosynthetic pathways have largely been characterized [27, 30, 97, 100–105] and have been shown to vary greatly under different conditions, such as under high and low mineral nutrition [32–38], under various light intensity, light quality, and photoperiod conditions [39, 40], across garlic varieties [41–43], and in response to various temperatures [39]. Cysteine and serine are precursors of alliin biosynthesis [20, 26], and allicin is derived from alliin via alliinase [69, 97, 106]. In this study, allicin concentrations in green garlic seedlings were higher than in blanched garlic seedlings (Fig. 1D). Interestingly, transcript levels for the DEGs *Asa1G01947*, *Asa0G01394*, *Asa1G04725*, and *Asa5G02119* encoding key genes involved in cysteine biosynthesis were also upregulated in green seedlings (Fig. 7). Similarly, most serine pathway–related genes and sulfur-related genes were upregulated in green seedlings. Moreover, nine genes and three genes encoding alliinase were upregulated and downregulated, respectively, in green seedlings (Fig. 7). The above findings thus provide support for the role of cysteine, serine, and sulfur-related metabolism in alliin biosynthesis. Our findings are also consistent with a previous report [26].

Different methods used for the extraction of secondary metabolites rich in sulfur from garlic tissue may affect the type, quantity, and quality of sulfur compounds obtained [21, 107, 108]. Using hydrodistillation and gas chromatography–mass spectrometry (GC–MS) methods, 16 volatile compounds in green seedlings and 14 volatile compounds in blanched seedlings were previously identified [21]. The same study determined that sulfur-containing compounds were extracted at a higher yield in blanched seedlings than in green seedlings. In sharp contrast, we determined that the allicin contents of green seedlings were higher than in blanched seedlings (Fig. 1D). Four possible reasons may explain these inconsistencies, such as 1) the garlic variety used, 2) extraction and determination methods, 3) cultivation methods, and 4) growth stages. First, extraction and determination methods were different between the two studies [21]. Furthermore, the previous study purchased garlic bulbs from the local food market and did not describe the garlic variety, the cultivation methods, or the growth stages in their experiments. In general, the market for blanched garlic seedlings and green garlic seedlings generally uses spreading varieties under soil cultivation, which are harvested after 20 to 30 days of growth. In this study, we grew the spreading variety ‘Jinxianghongsuan’ under hydroponic cultivation (using water only) and harvested samples after 14 days of growth, corresponding to the time when the nutrition provided by the garlic bulbs had just been exhausted. We reasoned that 14 days of growth would constitute the best time to study the regulation of garlic growth and gene expression by light or dark signals. A comparison of the two experiments shows that the quality of blanched seedlings can be improved by providing a nutrient solution. Hydroponic culture is an effective method to study the effects of nutrient elements on the quality of green and blanched garlic seedlings. This simple and effective method should offer a new way to cultivate green and blanched garlic seedlings with high levels of calcium, iron, and selenium.

Light, shade, and darkness all act as signals to regulate phytohormone levels, signaling pathways and transcription factors expression to affect plant development [39, 47, 48, 50, 82–85]. However, the mechanism by which light quality affects allicin synthesis remains unclear. Transcription factors contribute greatly to the regulation of plant growth and development and are also likely to play a role in allicin biosynthesis [26]. Major plant TF families such as bHLH, NAC, ETHYLENE RESPONSE FACTOR (ERF), MYB, and AP2 have been documented as important regulators in plant allicin biosynthesis [26]. In this study, genes encoding bHLH, NAC, ERF, and MYB family members were clearly differentially expressed between green and blanched seedlings. The light signal may control the expression of key genes in allicin synthesis by influencing these transcription factors, prompting us to select these differentially expressed TF genes for further analysis (Fig. 9).

### Analysis of plant–pathogen interaction pathways

Garlic is generally reproduced asexually through garlic cloves, which results in the accumulation of viruses [57]. The long history of co-evolution between garlic and its pathogenic microorganisms has gradually formed a complex network that balances plant defenses against pathogens and plant growth [54–56]. We examined the expression pattern of genes belonging to the plant–pathogen interaction KEGG pathway in green and blanched seedlings, which revealed both common and specific mechanisms associated with resistance to viruses. In green garlic seedlings, resistance mechanisms were associated with the differential expression of genes encoding WRKY TFs, leucine-rich repeat (LRR) receptor-like serine/threonine-protein kinases, serine/threonine-protein kinases, the disease resistance protein RESISTANT TO P. SYRINGAE2 (RPS2), 3-ketoacyl-CoA synthase, and EIX receptor, as well as BRs and jasmonic acid. Pathogen responses in blanched garlic seedlings were mainly associated with genes encoding heat shock proteins, the serine/threonine-protein kinase AVRPPHB SUSCEPTIBLE1 (PBS1), 3-ketoacyl-CoA synthase, and bHLH TFs. Indeed, many genes involved in the KEGG pathway plant–pathogen interaction were differentially expressed, with 152 DEGs in green seedlings and 55 DEGs in blanched seedlings (Table S12). It has also been reported in some studies that light can induce upregulated expression of genes involved in the KEGG pathway plant–pathogen interaction in different plants [109, 110]. Furthermore, we detected a significant enrichment for the KEGG pathways isoflavonoid biosynthesis, carotenoid biosynthesis, phenylpropanoid biosynthesis, and flavonoid biosynthesis, suggesting that they may be involved in the pathogen responses of green garlic seedlings. However, in blanched garlic seedlings, only the KEGG pathway flavone and flavonol biosynthesis associated with resistance mechanisms was enriched (Fig. 4). The response mechanisms to viruses or other stresses were thus more complex in green seedlings than in blanched seedlings.

MAPK signaling cascades involved in defense and disease resistance against pathogens have been extensively described; they mainly regulate the transcriptional activation of defense-related genes, induce the biosynthesis of plant antitoxins and cell wall thickening, hypersensitivity, stomatal closure, phytohormone biosynthesis, and bursts of reactive oxygen species [111–113]. In this work, DEGs were significantly enriched in the KEGG pathway MAPK signaling pathway-plant (ko04016). As shown in Fig. 6B, MAPK cascades were involved in defense and disease resistance in green garlic seedlings, via flg22, H_2_O_2_, phytohormone, and stomatal developmental pathways. Notably, these pathways counted more downregulated genes in green seedlings than the number of upregulated genes in blanched garlic seedlings. However, defense and disease resistance mechanisms against pathogens were similar in blanched and green garlic seedlings.

The cell wall is the first barrier between plants and pathogens, but endoglucanases and xyloglucan endotransglucosylase/hydrolases can loosen the cell wall by selective hydrolysis of cellulose and hemicellulose. Previous studies have reported that increased expression of the encoding genes can reduce tolerance against biotic stress in plants [114–116]. In this study, three endoglucanase genes and 28 xyloglucan endotransglucosylase/hydrolase genes were upregulated in blanched garlic seedlings, with another six xyloglucan endotransglucosylase/hydrolase genes being downregulated (Table S8, Fig. 8), suggesting that dark and light signals can affect the defense and disease resistance against pathogens in garlic seedlings by regulating cell wall biosynthesis and catabolism.

Overall, compared to blanched garlic seedlings, green garlic seedlings grown in the light exhibited the activation of more defense and disease resistance pathways, such as synthesizing large amounts of secondary metabolites, increased expression of genes involved in MAPK signaling pathway, and strengthening the cell wall, which may be associated with greater resistance against pathogens.

## Conclusions

According to our research, garlic seedlings quality and productivity can be impacted by dark and light signals. A comprehensive transcriptomic dataset analysis of garlic seedlings grown in light and darkness conditions was performed using RNA-seq technology, and a group of DEGs, which may regulate seedlings growth, allicin biosynthesis and defense and disease resistance through pairwise comparison analysis of DEGs between different light treatments, had been identified. This group included unigenes involved in phytohormone levels and signal transduction pathway, cell wall metabolism, secondary metabolites, MAPK signaling pathway and IFs. Overall, the resources generated by this study would lay foundation for revealing the molecular mechanism of how dark and light signals affect plant growth and the biosynthesis of compounds in garlic seedlings, as well as provide a theoretical basis for other *Allium* plants being grown in facility. Future studies will focus on improving the quality of blanched garlic seedlings by adjusting the composition of the nutritional solution in the later growth stage as well as light intensity to improve disease resistance of blanched garlic seedlings.

## Methods

### Plant materials

All trials were carried out with garlic bulbs stock plants from ‘Jinxianghongsuan’, an important and popular variety in China. All garlic bulbs were purchased from Jin Xiang Suan ye Co. Ltd. Green garlic seedlings and blanched garlic seedlings were grown at a local greenhouse facility for plant germplasm resources and genetic engineering, at Henan University (114°21′N, 34°47′W), China. All garlic bulbs were placed on a polyethylene tray floating on tap water for cultivation and growth, with a distance between seedlings of 2 × 2 cm. The green seedlings were grown in a day/night temperature cycle of 25/20 °C with a photoperiod of 16 h light/8 h darkness. To induce blanching, a set of garlic bulbs were cultivated in a breathable cardboard box covered with foil (to maintain complete darkness) in the same conditions as the green garlic seedlings. Change the water every three days. Samples were harvested after 14 days of growth in triplicates and stored in liquid nitrogen at –80 °C until further use. Due to the requirements of biological replicates, the seedlings from different three plants were sampled.

### Measurements of total allicin, soluble sugars, and cellulose contents

Green garlic seedlings and blanched garlic seedlings were grown for 14 days before collecting samples. Total cellulose was quantified colorimetrically using the phenol–sulfuric acid method as described [117]. Total soluble sugars contents were assessed by the anthrone method and the Coomassie Brilliant Blue method using an assay kit (Shanghai MLBIO Biotechnology Co. Ltd, Shanghai, China). Allicin contents were determined by the phenylhydrazone method as previously described [118]. All experiments were conducted in three biological replicates.

### Transcriptome analysis

Total RNA extraction from garlic seedlings, RNA-seq cDNA library preparation, and sequencing were carried out by Biomarker Technologies Company (Beijing, China). According to the manufacture’s protocol, total RNA from green garlic seedlings and blanched garlic seedlings was extracted with Trizol reagent and quantified using a Qubit® RNA Assay Kit on a Qubit®2.0 Fluorometer (Life Technologies, Carlsbad, CA, USA). RNA quality and integrity were assessed on an RNA Nano 6000 Assay Kit and an Agilent Bioanalyzer 2100 system (Agilent Technologies, Santa Clara, CA, USA). cDNA library construction was performed using the NEBNext® UltraTM RNA Library Prep Kit for Illumina® (NEB, San Diego, CA, USA). Six cDNA libraries (three biological replicate for green garlic seedlings, three biological replicate for blanched garlic seedlings, the samples from different three plants were sampled) were sequenced on an Illumina Hiseq™ 2500 platform (Biomarker Technologies Company, Beijing, China).

HISAT (Version: v2.1.0) [119] was used to filter and align the clean reads to the *Allium sativum* ‘Ershuizao’ reference genome [19]. Differential expression analysis was performed using DEseq2_EBseq with a false discovery rate (FDR) ≤ 0.05 and an absolute fold-change (FC) ≥ 2. GO and KEGG pathway [63–65] enrichment analysis of the differentially expressed genes (DEGs) was performed with the GOseq R package based on a Kolmogorov–Smirnov test [120] using KOBAS software [121].

### Real-time PCR analysis

The extraction of total RNA, cDNA synthesis, and RT-qPCR were performed as previously described [122]. The primers are listed in Supplementary Table 10. The RT-qPCR analysis of each sample was performed in triplicate. *GAPDH* (Gene id: Asa0G02401) was used as an internal reference, and the relative gene expression levels were calculated according to the 2^–ΔΔCT^ method [123].

### Statistical analysis

Statistical significance was assessed using SPSS software version 18.0 (SPSS Inc., Chicago, IL, USA) by *t*-test or one-way analysis of variance (ANOVA). Statistical significance is shown at *p* < 0.05 or *p* < 0.01.

## Supplementary Information

**Additional file 1.**

## Data Availability

RNA-Seq data generated in the study have been deposited in the National center for Biotechnology Information (NCBI) under the accession codes of Bio Project ID: PRJNA797535 (https:// www. ncbi. nlm. nih. gov/ bioproject/PRJNA797535).

## Abbreviations

DEGs: Differentially expressed genes
GO: Gene Ontology
KEGG: Kyoto Encyclopedia of Genes and Genomes
FPKM: Reads mapped per 1000 bp per million sequenced reads
TF: Transcription factor
qRT-PCR: Quantitative real-time PCR
