# Supplementary figures and images for: Comparative analysis of two kinds of garlic seedings: qualities and transcriptional landscape
Source: BMC Genomics. 2023 Feb 24;24:87. doi: 10.1186/s12864-023-09183-x (PMC9951544; doi:10.1186/s12864-023-09183-x)

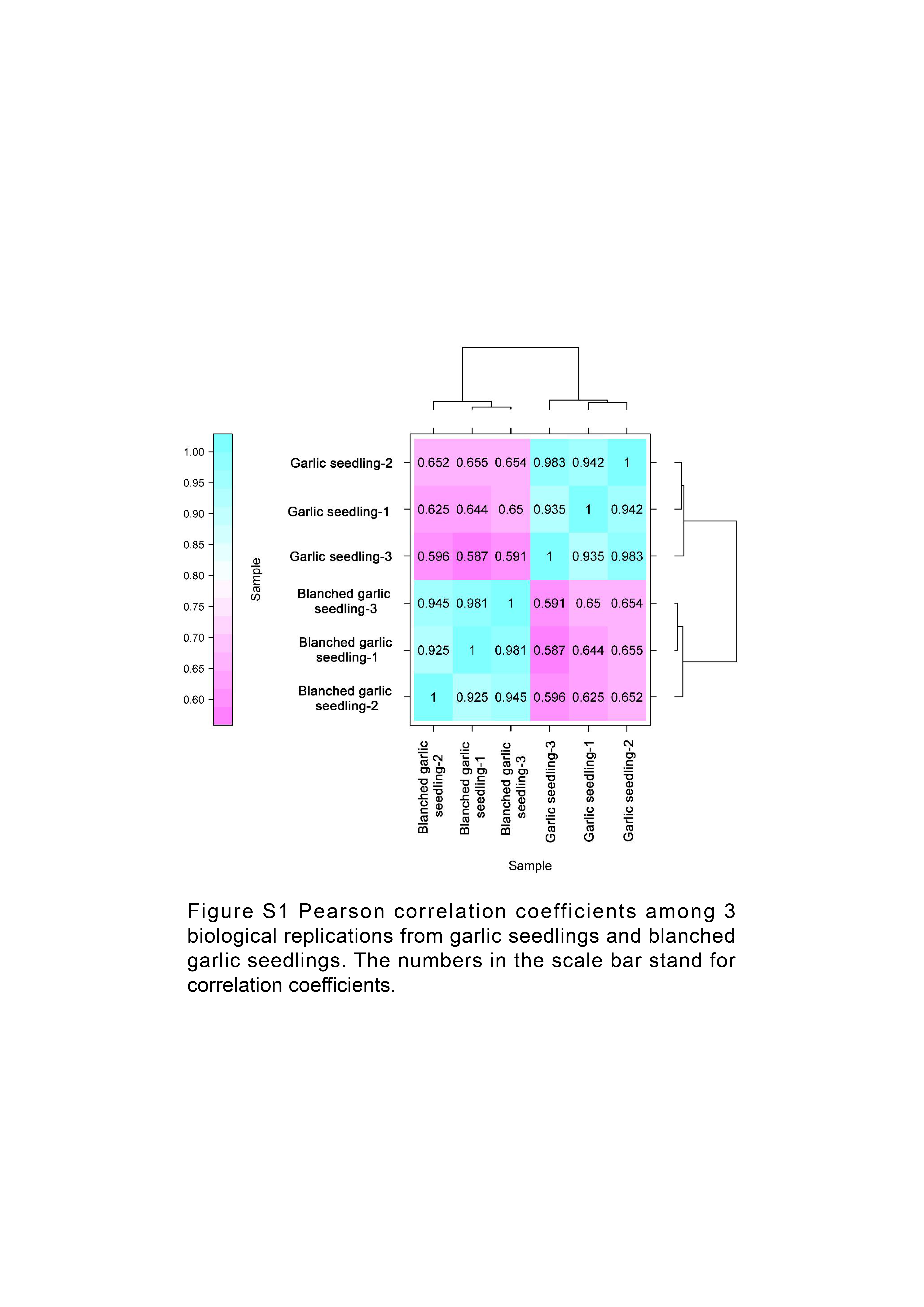

Supplement: Supplementary file 1 — Additional file 1. [file 12864_2023_9183_MOESM1_ESM.zip › Supplementary/Fig S1.tif]

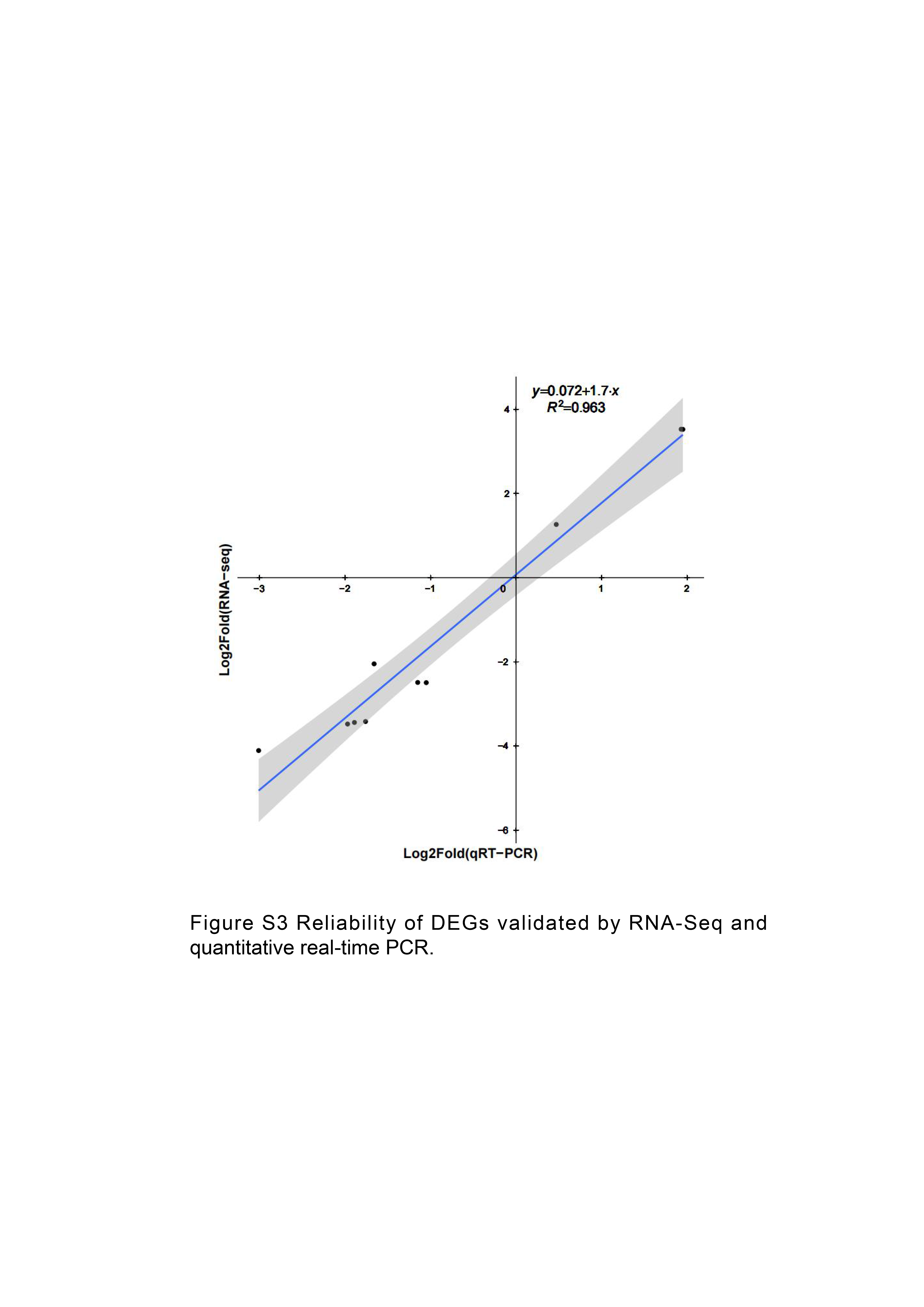

Supplement: Supplementary file 1 — Additional file 1. [file 12864_2023_9183_MOESM1_ESM.zip › Supplementary/Fig S3.tif]
